# The Impact of Ocean Acidification on the Functional Morphology of Foraminifera

**DOI:** 10.1371/journal.pone.0083118

**Published:** 2013-12-17

**Authors:** Nikki Khanna, Jasmin A. Godbold, William E. N. Austin, David M. Paterson

**Affiliations:** 1 Scottish Oceans Institute, School of Biology, University of St Andrews, St Andrews, Scotland; 2 Ocean and Earth Science, National Oceanography Centre, University of Southampton, Southampton, United Kingdom; 3 School of Geography & Geosciences, University of St Andrews, St Andrews, Scotland; 4 Scottish Association for Marine Science, Scottish Marine Institute, Oban, Scotland; 5 Ichron Limited, Northwich, United Kingdom; The Pennsylvania State University, United States of America

## Abstract

Culturing experiments were performed on sediment samples from the Ythan Estuary, N. E. Scotland, to assess the impacts of ocean acidification on test surface ornamentation in the benthic foraminifer *Haynesina germanica*. Specimens were cultured for 36 weeks at either 380, 750 or 1000 ppm atmospheric CO_2_. Analysis of the test surface using SEM imaging reveals sensitivity of functionally important ornamentation associated with feeding to changing seawater CO_2_ levels. Specimens incubated at high CO_2_ levels displayed evidence of shell dissolution, a significant reduction and deformation of ornamentation. It is clear that these calcifying organisms are likely to be vulnerable to ocean acidification. A reduction in functionally important ornamentation could lead to a reduction in feeding efficiency with consequent impacts on this organism's survival and fitness.

## Introduction

Elevated atmospheric concentrations of carbon dioxide (CO_2_), primarily driven by anthropogenic activity, are driving down the pH of the oceans [1]. It is estimated that the oceans have absorbed half of the total anthropogenic CO_2_ produced in the last 200 years, reducing oceanic pH by 0.1 units [1] and simultaneously affecting the carbonate ion concentration [2]. A further decrease of 0.3–0.5 pH units is predicted by 2100 [3]. One of the most significant implications is the likely reduction in calcifying capacity of marine organisms [4], [5]. The experimental responses of organisms that produce carbonate structures have however been variable depending on the type and length of exposure [6], [7].

Foraminifera are amoeboid protozoa that constitute one the most widespread and diverse groups of shelled microorganisms in the modern oceans. They occur in planktic and benthic habitats from the intertidal coastal habitats to the deep sea [8]. The majority of benthic foraminifera build their tests (shells) with calcium carbonate (CaCO_3_) [9] playing an important role in the carbon cycle of intertidal estuarine sediments [10].

**Figure 1:**
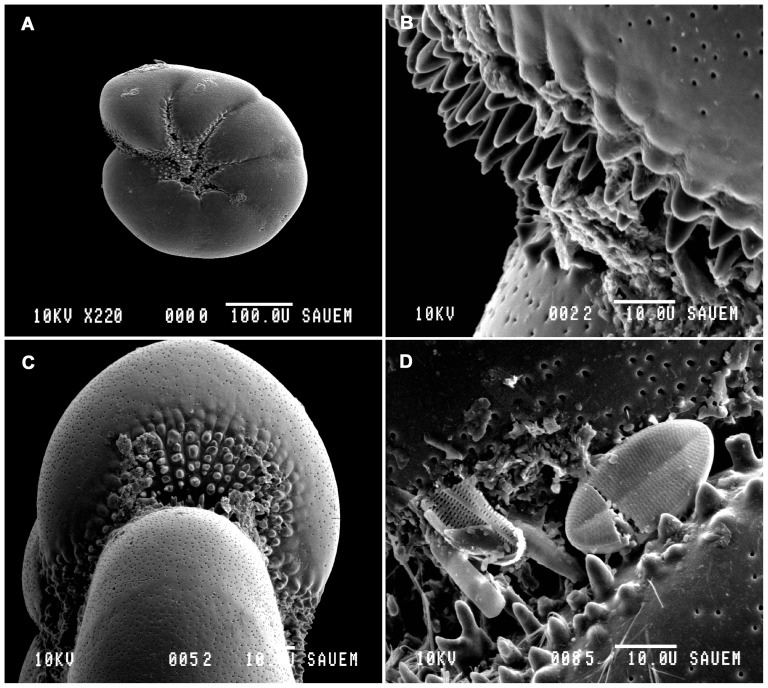
Scanning electron micrographs of *Haynesina germanica*. (A) Scanning electron micrograph of typical test, side view. (B) Higher magnification view of apertural region, showing tubercles and teeth lining the aperture. (C) Scanning electron micrograph of typical test, apertural view. (D) Higher magnification view of apertural region. Note impaled diatom (*).

The evolutionary and ecological success of shelled foraminifera depends on the functional significance of the test [11], [12]. The test provides shelter and protection from predators, assistance in cell growth, aids in reproduction, acts as a receptacle for excreted matter, and provides buoyancy control [13]–[15]. There are also morphological features and ornamentation on the test that are of functional importance. For example, tubercles and teeth in the apertures (Fig. 1) of some species are important in feeding and serve to break up aggregates of food and detritus [16]. Tubercles are protrusions that border the chamber margins while teeth are similar protrusions that are found adjacent to the aperture, occupying part of the apertural space [17]. Hamm et al. [18] speculated that diatom grazers are likely to have evolved specialised tools to break open frustules. Austin et al. [12] documented extracellular cracking and removal of diatom contents, aided by the presence of ‘tooth-like’ dentition in *Haynesina germanica* (Ehrenberg).

To date, researchers have examined ocean acidification impacts on benthic foraminifera via test weight, thickness and growth rate [19]–[21]. However, there is currently no information on how foraminiferal test functional morphology might respond to ocean acidification.

Here we present ultrastructural observations using scanning electron microscopy of cultured *H. germanica* to examine the effect of different levels of atmospheric CO_2_ on test morphology, focussing on the apertural region known to have functional ornamentation [12].

## Materials and Methods

Sediment was collected from the Ythan Estuary, N.E. Scotland (57°20′N, 01°57′W), in December 2010. No specific permissions were required for sediment sampling at this location and the sampling did not involve endangered or protected species. Sieved sediment (500 µm), was added to acrylic mesocosms (12×12×33 cm) to a depth of 12 cm and 20 cm of overlying seawater. Mesocosms were exposed to a seasonally varying light cycle and maintained at 10°C (average local conditions). Seawater pH was controlled by bubbling air with a known concentration of CO_2_ (380, 750, 1000 ppm) into each mesocosm [22]. Seawater pH, temperature and salinity were monitored weekly and atmospheric CO_2_ was maintained at 380, 750 or 1000 ppm (Table S1). After 36 weeks of exposure, surface scrapes of 1–2 mm depth were collected from each of the treatments for the isolation of living benthic foraminifera.

The surface scrapes were fixed in ethanol and stained with rose Bengal (to distinguish live individuals). After 24 h, samples were washed over a 63 µm sieve and the coarse fraction dried (<40°C). 15 live adult specimens of *Haynesina germanica* (300–400 µm), five from each CO_2_ treatment, were selected using a fine paintbrush under an Olympus® SZ stereo-microscope and dry-stored on micropalaeontological slides prior to imaging with a scanning electron microscope (SEM). The foraminiferal specimens were mounted onto SEM stubs using double-sided adhesive tabs. Samples were prepared with an Emscope® SC 500 sputter coater and imaged with a scanning electronic microscope (Joel® JSM-35CF SEM). For each specimen, the number of teeth in a 20 µm^2^ region of the apertural area were counted and measured (SemAfore®) for maximum length and width (Table 1). Once assumptions were confirmed, a one-way ANOVA was used to determine the effects of CO_2_. Analyses were performed using the statistical programming software ‘R’ [23].

**Table 1:** Summary of main morphological features at each CO_2_ treatment.

CO_2_ Treatment (ppm)	average number of *“teeth” (mean*±*SD)*	average length of *“teeth” (mean*±*SD)*	average width of *“teeth” (mean*±*SD)*	observational notes on *“teeth”*	test surface observations	number of diatom frustules in apertural region
380	15.20±2.49 (n = 76)	6.53±2.13	3.27±1.00	Teeth predominantly conical in shape	Test surfaces smooth with little evidence of dissolution	7
750	10.80±3.96 (n = 54)	5.26±1.79	3.27±1.16	Teeth conical in shape. A number of broken teeth also observed.	Some small areas of dissolution and pitting evident.	2
1000	6.60±2.51 (n = 33)	5.01±1.94	3.99±1.97	Teeth tended to be fewer and generally more rounded in shape. Of those counted a number were broken or deformed.	Dissolved patches more extensive and numerous in addition to visible cracking	0

## Results

SEM imaging of *Haynesina germanica* revealed differences between specimens cultured in each of the CO_2_ treatments. There was a statistically significant difference in the average number (df = 2, F = 9.8369, p<0.05), length (df = 2, F = 9.6467, p<0.0001) and width (df = 2, F =  4.0073, p<0.05) of teeth.

**Figure 2:**
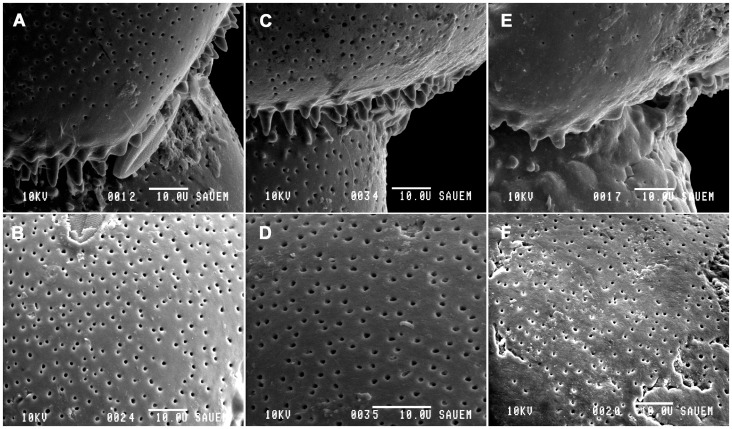
Scanning electron micrographs of *Haynesina germanica* following exposure to each of the CO_2_ treatments. SEM images of specimens taken at 380(A & B), 750 ppm (C & D) and 1000 ppm (E & F). (A) Side view of apertural region showing numerous sharp tubercles. Note diatom impaled on ornamentation (*). (B) Scanning electron micrograph of test surface of specimen 2A. (C) Side view of apertural region, showing tubercles and teeth. (D) Scanning electron micrograph of test surface of specimen 2C. (E) Side view of apertural region. Note the distinct absence of the numerous conical tubercles present in 2A. (F) Scanning electron micrograph of test surface of specimen 2E. Note surface dissolution and cracking damage.

. 2A). Several specimens also had diatoms impaled on teeth close to the apertural region. In addition, the test surface was smooth and unblemished (Fig. 2B).

At 750 ppm, specimens displayed similar shell appearance to those observed at 380 ppm (Fig. 2C). The teeth were of a similar size and shape but in many cases there was a reduction in overall number (10.80±3.96) in comparison to the control (15.20±2.49). The test surfaces also appeared smooth although some signs of cracking were evident (Fig. 2D).

At the highest CO_2_ treatment (1000 ppm), there were marked signs of dissolution and deformation features on multiple individuals in comparison to those cultured at 380 and 750 ppm. Dissolution was evident on the test surface (Fig. 2E, F) and in many cases the teeth were completely absent (Fig. 2E). In those specimens from the high CO_2_ treatment that retained some ornamentation, the shape and appearance was highly irregular. Teeth were less conical and more rounded in shape. In addition, the entire test surface appeared to be partially dissolved with evidence of cracking (Fig. 2F), which was entirely absent from control specimens.

## Discussion

Measurement data demonstrate that test ornamentation of functional importance for *Haynesina germanica* is sensitive to decreasing seawater pH. Under ambient conditions, the test surface of *H. germanica* is smooth with the primary openings along the base of the apertural face being obscured externally by teeth that are usually conical in shape. Foraminifera maintained at ambient CO_2_ levels had tubercles and teeth that were numerous and very well developed in both the apertural and umbilical regions. At higher CO_2_ levels the functional ornamentation of the apertural area was vastly reduced and the extent to which these apertural and umbilical features were dissolved or damaged makes the foraminifera almost unrecognizable as *H. germanica* (Fig. 2).

One of the keys to evolutionary success of foraminifera are the finely tuned relationships they have developed to exploit the food resources of their community [24]. Bernard and Bowser [16] document the significance of foraminiferal test functional morphology in species known to sequester chloroplasts, and showed that the external test ornamentations facilitate separation of chloroplasts from algal prey. As particles are moved past the tubercles, they are sorted by size, where larger fragments become disaggregated in preparation for later ingestion. Austin et al. [12] showed a direct relationship between fracturing in diatoms and feeding sequestration by *H. germanica*.

Test ornamentation, notably around the apertural area, is therefore crucial for feeding and chloroplast acquisition in *H. germanica*. Dissolution of these features at anticipated future levels of atmospheric CO_2_ will therefore have a direct negative impact on the long-term fitness and survival on these organisms through a reduction in feeding efficiency.

The potential for deformation, dissolution and in some cases absence of these functionally important feeding structures, may result in a shift in competitive advantage towards non-calcifiers in the benthic foraminiferal community under conditions of enhanced ocean acidification. This shift in community structure is likely to be further enhanced as a result of weakened tests under high-CO_2_ scenarios, making calcifying foraminifera such as *H. germanica* more vulnerable to predators. Such shifts in community structure will have significant knock-on effects for trophic dynamics, carbon cycling and ecosystem productivity.

## Supporting Information

Table S1 **Seawater measurements from experimental mesocosms.** The measured values of temperature, salinity and pH (NBS scale) and total alkalinity (A_T_) were used to calculate the values of dissolved inorganic carbon (DIC), pCO_2_, saturation states of calcite (Ω_calcite_) and aragonite (Ω_aragonite_), bicarbonate (HCO_3_) and carbonate concentration (CO_3_^2−^) using CO_2_Calc.
(PDF)
