# Correction: The Impact of Ocean Acidification on the Functional Morphology of Foraminifera

**DOI:** 10.1371/annotation/db02085d-b611-4071-ad7d-8d896ab01168

**Published:** 2014-01-29

**Authors:** Nikki Khanna, Jasmin A. Godbold, William E. N. Austin, David M. Paterson

In the second paragraph of the Results section, an error occurred during production in which a sentence was deleted. 

The first sentence of this paragraph currently reads: . 2A).

It should read: "Control specimens of *H. germanica* maintained at 380 ppm possessed teeth that were both numerous and conical in shape (Fig. 2A)."

