# Supplementary material for: The Impact of Ocean Acidification on the Functional Morphology of Foraminifera
Source: PLoS One. 2013 Dec 17;8(12):e83118. doi: 10.1371/journal.pone.0083118 (PMC3866275; doi:10.1371/journal.pone.0083118)
Supplement: Table S1 — Seawater measurements from experimental mesocosms. The measured values of temperature, salinity and pH (NBS scale) and total alkalinity (AT) were used to calculate the values of dissolved inorganic carbon (DIC), pCO2, saturation states of calcite (Ωcalcite) and aragonite (Ωaragonite), bicarbonate (HCO3) and carbonate concentration (CO3 2−) using CO2Calc. (PDF) [file pone.0083118.s001.pdf]

Table S1

Month	Target CO_2 (ppm)	Temperature (°C)	Salinity	pH_{NBS}	A_T (µmol kg^{-1})	DIC (µmol kg^{-1})	pCO_2 (µAtm)	$\Omega_{Calcite}$	$\Omega_{Aragonite}$	HCO_3^-	CO_3^{-2}
1	380	10.27 ± 0.09	33.40 ± 0.06	8.13 ± 0.056	2665 ± 19	2479 ± 45	506 ± 71	3.54 ± 0.38	2.25 ± 0.24	2310 ± 58	147 ± 16
1	750	10.10 ± 0.00	33.60 ± 0.06	8.04 ± 0.021	2661 ± 12	2518 ± 19	633 ± 38	2.89 ± 0.12	1.83 ± 0.07	2370 ± 22	120 ± 5
1	1000	10.10 ± 0.06	33.53 ± 0.13	7.78 ± 0.015	2913 ± 221	2867 ± 224	1331 ± 142	1.82 ± 0.10	1.15 ± 0.06	2732 ± 214	76 ± 4
3	380	9.58 ± 0.08	33.28 ± 0.09	8.10 ± 0.032	2836 ± 38	2665 ± 51	582 ± 53	3.42 ± 0.18	2.17 ± 0.12	2497 ± 56	142 ± 8
3	750	9.48 ± 0.05	33.25 ± 0.05	7.92 ± 0.043	2987 ± 55	2886 ± 39	953 ± 82	2.53 ± 0.29	1.60 ± 0.18	2738 ± 32	105 ± 12
3	1000	9.55 ± 0.05	33.13 ± 0.05	7.76 ± 0.011	2930 ± 59	2893 ± 61	1377 ± 56	1.74 ± 0.04	1.10 ± 0.02	2759 ± 58	72 ± 2
5	380	9.93 ± 0.26	33.37 ± 0.24	7.99 ± 0.083	3089 ± 95	2949 ± 61	845 ± 159	3.15 ± 0.59	2.00 ± 0.38	2781 ± 46	131 ± 25
5	750	10.23 ± 0.19	32.50 ± 0.50	7.89 ± 0.041	2997 ± 32	2911 ± 26	1057 ± 93	2.36 ± 0.21	1.49 ± 0.13	2767 ± 24	98 ± 8
5	1000	10.17 ± 0.12	32.77 ± 0.12	7.74 ± 0.014	3083 ± 31	3053 ± 31	1550 ± 55	1.76 ± 0.06	1.11 ± 0.04	2912 ± 30	73 ± 2
6	380	9.83 ± 0.08	31.85 ± 0.16	8.04 ± 0.033	3057 ± 135	2913 ± 132	749 ± 74	3.19 ± 0.24	2.02 ± 0.15	2748 ± 126	132 ± 10
6	750	9.55 ± 0.09	31.53 ± 0.14	7.90 ± 0.004	3163 ± 93	3076 ± 89	1068 ± 26	2.45 ± 0.10	1.55 ± 0.06	2927 ± 84	101 ± 4
6	1000	9.58 ± 0.05	31.40 ± 0.06	7.76 ± 0.009	3217 ± 72	3185 ± 72	1527 ± 44	1.85 ± 0.06	1.17 ± 0.04	3040 ± 69	76 ± 2
9	380	9.70 ± 0.06	30.07 ± 0.03	8.01 ± 0.000	2743 ± 98	2631 ± 95	725 ± 25	2.58 ± 0.10	1.62 ± 0.06	2493 ± 90	105 ± 4
9	750	9.77 ± 0.09	30.33 ± 0.03	7.85 ± 0.014	2993 ± 60	2933 ± 58	1161 ± 36	2.05 ± 0.08	1.29 ± 0.05	2796 ± 55	84 ± 3
9	1000	9.70 ± 0.09	30.47 ± 0.03	7.72 ± 0.005	2699 ± 19	2685 ± 18	1430 ± 11	1.39 ± 0.02	0.88 ± 0.01	2563 ± 17	57 ± 1
